# Metabolic reprogramming by KSHV in cellular transformation

**DOI:** 10.1186/2049-3002-2-S1-P88

**Published:** 2014-05-28

**Authors:** Ying Zhu, Suzane Ramos da Silva, Shou-Jiang Gao

**Affiliations:** 1 Department of Molecular Microbiology and Immunology, Keck School of Medicine, University of Southern California, Los Angeles, CA, USA

## 

Oncogenic Kaposi’s sarcoma-associated herpesvirus (KSHV) is causally linked to several human cancers including Kaposi’s sarcoma and primary effusion lymphoma. Despite intensive studies, little is known about how KSHV alters cellular metabolism during cellular transformation. Using a novel model of KSHV-induced cellular transformation of primary rat embryonic metanephric mesenchymal stem cells (MM), we found that KSHV-transformed MM (KMM) cells did not require glucose for growth and formation of colonies in soft agar. Consistent with these results, KMM cells had lower levels of glucose consumption, lactate production, oxygen consumption and intracellular ATP levels. Compared to MM cells, KMM cells had lower levels of glucose transporters GLUT1 and GLUT3. Genetic deletion of KSHV-encoded microRNA cluster or vFLIP gene from the viral genome, which resulted in reduced NF-κB activity, significantly increased glucose consumption and lactate production, and sensitized the cells to glucose deprivation. Accordingly, inhibition of the NF-κB pathway with either RelA siRNAs or inhibitor JSH-23 dramatically increased glucose consumption and lactate production. Thus, the insensitivity of KMM cells to glucose is mediated by the heightened NF-κB activity. In contrast to the insensitivity to glucose, KMM cells were sensitive to glutamine deprivation. Glucose deprivation further increased glutamine consumption of KMM cells. Inhibition of glutamine metabolism with amino oxyacetate (AOA), an inhibitor of glutamate-dependent transaminases, or epigallocatechin gallate (EGCG), an inhibitor of glutamate dehydrogenase (GDH) mimicked the effect of glutamine deprivation and effectively suppressed cell growth and formation of colonies in soft agar. Addition of nonessential amino acid (NEAA) mixture or asparagine alone was sufficient to restore the growth of KMM cells upon glutamine deprivation while dimethyl α-ketoglutarate only had partial effect, indicating that glutamine not only provided a carbon source to fuel the tricarboxylic acid (TCA) cycle but also supplied nitrogen for biosynthesis. Taken together, these results indicate that KMM cells depend neither on aerobic glycolysis nor on oxidative phosphorylation but instead on glutamine to support cell growth and transformation. Our findings also suggest that metabolic reprogramming might have a critical role in KSHV-induced cellular transformation.

